# Feasibility and preliminary efficacy of the ‘HEYMAN’ healthy lifestyle program for young men: a pilot randomised controlled trial

**DOI:** 10.1186/s12937-017-0227-8

**Published:** 2017-01-13

**Authors:** Lee M. Ashton, Philip J. Morgan, Melinda J. Hutchesson, Megan E. Rollo, Clare E. Collins

**Affiliations:** 1 School of Health Sciences, Faculty of Health and Medicine, Priority Research Centre in Physical Activity and Nutrition, University of Newcastle, Callaghan, Australia; 2 School of Education, Faculty of Education and Arts, Priority Research Centre in Physical Activity and Nutrition, University of Newcastle, Callaghan, Australia

**Keywords:** Behavioural health, Process evaluation, Physical activity, Diet, Mental health, Intervention, Young men

## Abstract

**Background:**

In young men, unhealthy lifestyle behaviours can be detrimental to their physical and/or mental health and set them on a negative health trajectory into adulthood. Despite this, there is a lack of evidence to guide development of effective health behaviour change interventions for young men. This study assessed the feasibility and preliminary efficacy of the ‘HEYMAN’ (Harnessing Ehealth to enhance Young men’s Mental health, Activity and Nutrition) healthy lifestyle program for young men.

**Methods:**

A pilot RCT with 50 young men aged 18–25 years randomised to the HEYMAN intervention (*n* = 26) or waitlist control (*n* = 24). HEYMAN was a 3-month intervention, targeted for young men to improve eating habits, activity levels and well-being. Intervention development was informed by a participatory research model (PRECEDE-PROCEED). Intervention components included eHealth support (website, wearable device, Facebook support group), face-to-face sessions (group and individual), a personalised food and nutrient report, home-based resistance training equipment and a portion control tool. Outcomes included: feasibility of research procedures (recruitment, randomisation, data collection and retention) and of intervention components. Generalized linear mixed models estimated the treatment effect at 3-months for the primary outcomes: pedometer steps/day, diet quality, well-being and several secondary outcomes.

**Results:**

A 7-week recruitment period was required to enrol 50 young men. A retention rate of 94% was achieved at 3-months post-intervention. Retained intervention participants (*n* = 24) demonstrated reasonable usage levels for most program components and also reported reasonable levels of program component acceptability for attractiveness, comprehension, usability, support, satisfaction and ability to persuade, with scores ranging from 3.0 to 4.6 (maximum 5). No significant intervention effects were observed for the primary outcomes of steps/day (1012.7, 95% CI = −506.2, 2531.6, *p* = 0.191, *d* = 0.36), diet quality score (3.6, 95% CI = −0.4, 7.6, *p* = 0.081, *d* = 0.48) or total well-being score (0.4, 95% CI = −1.6, 2.5, *p* = 0.683, *d* = 0.11). Significant intervention effects were found for daily vegetable servings, energy-dense, nutrient-poor foods, MVPA, weight, BMI, fat mass, waist circumference and cholesterol (all *p* < 0.05).

**Conclusions:**

The HEYMAN program demonstrated feasibility in assisting young men to make some positive lifestyle changes. This provides support for the conduct of a larger, fully-powered RCT, but with minor amendments to research procedures and intervention components required.

**Trial registration:**

Australian New Zealand Clinical Trials Registry ACTRN12616000350426.

**Electronic supplementary material:**

The online version of this article (doi:10.1186/s12937-017-0227-8) contains supplementary material, which is available to authorized users.

## Background

Young men aged 18–25 years, experience a key transitional phase as they move from adolescence to adulthood. For many, this time is marked by major life changes including moving away from the family home, starting and completing further education, beginning employment or unemployment, co-habiting with peers or a partner, getting married and/or becoming a parent [1, 2]. Such transitions can adversely impact on health-related behaviours, including greater use of alcohol [3], poor eating habits [4, 5] and reduced physical activity [6]. This is a concern as habits in young adulthood commonly track into mid-adulthood [7] and worsen [8]. For instance; the Coronary Artery Risk Development in Young Adults (CARDIA) prospective cohort study (*n* = 3538) found that 75% of young adults aged 18–30 years either reduced the number of healthy lifestyle factors (i.e., non-smoking, low alcohol, healthy diet, active, or healthy BMI) or remained unchanged when followed-up 20 years later in middle-age [8]. If adverse behaviours continue or escalate they can be detrimental to the physical and/or mental health of young men and set them on an adverse health trajectory as they progress through adulthood [9–13]. Therefore, young adulthood is an ideal time to target improvements in these health-related behaviours in order to prevent or delay serious mental health problems [14] and future chronic disease risk such as cardiovascular disease [9], hypertension [15] and type 2 diabetes [16].

Recruiting, engaging and retaining young men into health-related interventions is an important yet challenging aspect of health research [17–19]. A number of reasons have been suggested regarding challenges to engaging young men including; perceived irrelevance given current life-stage [20], less likely to live in a fixed location, long-term [21], competing time demands which take priority (i.e., study, work, socialising, relationships, family obligations and/or parenthood) [21]. In addition, previous health programs’ have failed to account for the sociocultural values and preferences of young men in informing recruitment strategies and developing intervention components [22].

Difficulties associated with recruitment and retention may explain why young men are under-represented in health programs and why there is a lack of evidence to guide development of effective health-related interventions for young men [23, 24]. The current evidence base is predominantly made up of health-related interventions that include both sexes [23] and all ages [25, 26], but the heterogeneity in psychological, social, and physical differences between sexes and age groups, highlight the need for gender and age-specific health research and behavioural programs [27]. A recent systematic review of SNAPO (Smoking, Nutrition, Alcohol, Physical activity or Obesity) interventions in exclusively young men [24] found few interventions targeting young men (*n* = 10) and over half (6 out of 10) demonstrated significant positive short-term intervention effects. However, the review highlighted various limitations of studies including; only short-term outcomes reported, high risk of bias and difficulties in reaching and/or retaining this population group. Also none of the studies were specifically targeted or tailored to young men. The review concluded that more high quality studies are required that include young men in program design in order to personalise programs to their needs, interests and barriers, and to improve understanding of how to successfully engage them in effective health-behaviour change interventions.

A process evaluation of such studies can obtain vital perspectives from young men and is an integral component of intervention research to inform the design and implementation of future personalised interventions for this demographic [28]. As there is limited evidence on the effectiveness of health-related interventions in young men [24], a detailed process evaluation may help to identify and understand participants views of the program, how participants engage with and use the different intervention components and which treatment modalities are feasible and acceptable to young men [28]. In particular, process evaluation results can provide valuable insights into why an intervention fails or has unexpected outcomes or unintended consequences, or why a successful intervention works and how it can be optimised for a future RCT [29]. Understanding these aspects can help to overcome the difficulties apparent with reaching and retaining young men [23]. Therefore, the aims of the current study were to:

1. Evaluate the feasibility of a targeted healthy lifestyle program for young adult men aged 18–25 years.
2. Estimate the treatment effect of HEYMAN on improving objective physical activity levels (steps/day), diet quality and subjective well-being (primary outcomes in subsequent RCT) and other lifestyle, psychological, anthropometric and physiological measures (secondary outcomes in the subsequent RCT).

## Methods

### Study design

This was an assessor blinded, two-arm pilot randomised controlled trial (RCT) addressing feasibility and preliminary efficacy of the 3 month HEYMAN program. Following baseline measurement, young men were individually randomised to the HEYMAN group (commenced HEYMAN intervention immediately) or the waitlist control group (started HEYMAN after a 3-month delay). The trial was registered with the Australian New Zealand Clinical Trials Registry, Number ACTRN12616000350426. The design, conduct and reporting adhered to the guidelines as outlined by Thabane and colleagues [30]. The checklist is an adapted version of Consolidated Standards of Reporting Trials (CONSORT) guidelines [31] specifically for pilot studies.

### Intervention development

HEYMAN (**H**arnessing **E**health to enhance **Y**oung men’s **M**ental health, **A**ctivity and **N**utrition) is a multi-component targeted healthy lifestyle program, specifically for young men (aged 18–25 years) to improve eating habits, activity levels and overall well-being. The development of HEYMAN is based on guidance from a community based participatory research model; PRECEDE-PROCEED [32]. This model includes the target audience in developing the intervention to enhance program effectiveness and ensure that their individual needs and interests are accounted for, a strategy which should also improve reach, retention and engagement of young men [33, 34]. To align with the PRECEDE aspects of the model [32], a number of steps were taken to understand the social, epidemiological, behavioural and environmental assessments for this population group when developing HEYMAN. Formative research with young men was conducted to identify perceived motivators and barriers for healthy eating and physical activity [17, 35] and to identify their preferences for intervention content and delivery medium [36]. In addition, the program was informed by best practice guidelines for diet [37] and physical activity [38], theoretical guidelines from an integrated framework of Social Cognitive Theory (SCT) [39] and Self Determination Theory (SDT) [40], and evidence from effective health-related interventions in this population [23, 24]. See Additional file 1 for a full list of HEYMAN components and their alignment with the participatory responses from the formative work and with behaviours change strategies from SCT and/or SDT.

### Ethics

This study was approved by the University of Newcastle Human Research Ethics Committee (Approval number: H-2015-0445). Written informed consent was obtained from all subjects. Participants were offered a $AU10 gift voucher at baseline and follow up measurement sessions to cover travel expenses.

### Participants and recruitment

The HEYMAN study was conducted in young adult males (aged 18–25 years) from the Hunter region of New South Wales, Australia recruited via flyers distributed around the local university, technical colleges, workplaces, sports clubs and a barber shop. Information on the study was also advertised via posts on social media (Facebook and Twitter), which were shared on pages of the student researcher, local university, technical college, Hunter Medical Research Institute and local newspaper. In addition, a media release, with information appearing via the local newspaper, magazines and radio stations. Young men who took part in previous participatory research [17, 35] and who indicated an interest in being contacted via e-mail about future health programs were also invited to participate.

Participants were screened for eligibility via an online survey using a standardised protocol. Those eligible were required to self-report dietary and physical activity behaviours that failed to meet national recommendations [37, 38] and have access to an electronic device with e-mail and internet facilities. The program was designed to ensure that young men with existing health conditions were not excluded. All young men wishing to enrol completed a pre-exercise screener and the K-10 psychological distress scale [41]. Those answering ‘Yes’ to any question on the exercise screener and/or scored ≥30 on the K-10 psychological distress scale were advised to see their GP to obtain approval to participate in the program. A full list of the Eligibility criteria are outlined in Table 1.

**Table 1:** Inclusion and exclusion criteria for the HEYMAN program

Inclusion criteria	Exclusion criteria
• Male	• Self-reported meeting national recommendations for fruit and vegetable intakes (Based on age/sex recommendations: men aged 18 = 5 vegetables and 2 fruit, men aged 19–25 = 6 vegetables and 2 fruit daily) [74]
• Aged 18 to 25 years	• Self-reported meeting physical activity recommendations (moderate-intensity PA for 300 min or more per week or vigorous-intensity PA for 150 min or more per week or combined moderate and vigorous physical activity (MVPA) of 300 min or more per week) [38]
• Available for assessment sessions	• Currently participating in an alternative healthy lifestyle program.
• Access to a computer or tablet or smartphone with e-mail and Internet facilities	• History of major medical problems (such as heart disease or diabetes that requires insulin injections) that had not been granted GP approval to participate.^a^
	• Reported psychological distress and no GP approval (or associated expert) to participate^b^
	• Diagnosed with an eating disorder
	• Non-English speaking
	• Disability (e.g. physical/mobility disability, sight or hearing impairment) that precluded participation

^a^Those answering ‘yes’ to any of the conditions in the pre-medical exercise screener required GP approval to participate

^b^Those with a score of ≥30 on the K-10 psychological distress scale required GP approval to participate

### The HEYMAN intervention group

A detailed description of all intervention components are available in Additional file 1. In brief, young men randomised to the HEYMAN group received the following seven program components;

1. A responsive website that served as a ‘resource library’ housing relevant information and resources, including fact sheets from best practice guidelines, support videos (e.g. short cooking videos and demonstration of Gymstick™ exercises) and recommended mobile applications for improving eating habits, physical activity, reducing alcohol intake or coping with stress;
2. A Jawbone™ wearable physical activity tracker with associated mobile phone application (UP app) to assist in goal setting and self-monitoring of key health behaviours;
3. One-hour weekly face to face sessions at the university (11x group based and 1x individual). Sessions were delivered by two male researchers from the same age demographic (one was a qualified P.E. teacher, undertaking a PhD in Education and the other was a PhD candidate in Nutrition and Dietetics). Group based sessions took place on Thursday evenings (18:00–19:00 pm), with 40 min allocated for the practical exercise activities focusing on aerobic (e.g., team based recreational games) and strength exercises (e.g., High Intensity Interval Training). Also ten minutes were allocated for healthy eating education (e.g., meal planning and meal ideas for quick, cheap and healthy meals) and a designated 10 min for helping with stress and well-being, including a mixture of practical (e.g., mindfulness based stress reduction) and theoretical (e.g., problem solving strategies to address key issues apparent in young men, i.e., lack of money) components. The individual session took place in week three of the program and provided personalised feedback from a food and nutrient report (see below), and from the Jawbone physical activity data. From this personal tailored goals were set. All sessions were designed to address the participatory responses and used behaviour change strategies from the SCT and SDT.
4. Personalised food and nutrient report comparing intakes to Australian food and nutrient recommendations [37]. Data were calculated from the Australian Eating Survey food frequency questionnaire (FFQ) which was completed online at baseline and based on the participants’ eating habits over the previous six months. This feedback report was given to participants and discussed in the individualised session (week 3) and used to set personal tailored goals for dietary improvements;
5. A private Facebook discussion group to facilitate social support, send reminders for upcoming face-to-face sessions and send notifications for new material added to the website;
6. A Gymstick™ resistance band, for home-based strength training with linked routines available on the website
7. A TEMPlate™ dinner disc to guide main meal portion size for main meal components.

Participants were provided with the intervention materials at baseline and instructed to use them throughout the 3-month intervention period.

### The Waitlist control group

Control participants were asked to continue their usual lifestyle for 3 months and offered the HEYMAN program once follow-up assessments were completed.

### Data collection

Young men were measured at baseline and at 3 months in an anthropometry laboratory at the University of Newcastle, NSW, Australia. All measurements were performed by trained research assistants who were blinded to group allocation. Questionnaires were completed online prior to sessions.

### Outcomes

#### Feasibility

The primary outcomes for this pilot trial were feasibility of research procedures (recruitment, randomisation, data collection and retention) and of the intervention components (program usage, attractiveness, comprehension, usability, support, satisfaction and ability to persuade).

Recruitment was assessed during the eligibility screening survey by asking young men to report where they had heard about the program and also measured by the numbers interested versus those eligible. Retention was assessed as attendance at the 3-month follow-up measurements and completion of online questionnaires. Acceptability of randomisation was assessed by asking participants to rank overall satisfaction with the group allocation on a 5-point Likert scale from very satisfied (=5) to very unsatisfied (=1). Acceptability of data collection was estimated from the percentage of young men who completed all objective and self-report measures at baseline and follow-up.

Program component use was objectively tracked, including total number of website visits with average number of pages/tabs viewed and average duration of each visit (using Google™ analytics data); total number of views of the featured videos (using YouTube™ analytics data), Facebook discussion forum posts and attendance at face-to-face sessions. For program components that could not be objectively measured, participants were asked to report their frequency of use as part of the process evaluation questionnaire, with response options matched with the recommended frequency of use for each intervention component. For example, participants were instructed to use the Gymstick™ resistance band on two days per week and thus the response options ranged from “More than once per day” to “Never”. The recommended frequency for use for each of the intervention components are outlined in Additional file 1.

Attractiveness, comprehension, usability, support, satisfaction and ability to persuade of the HEYMAN intervention components were assessed by a post-program process evaluation survey, developed by the research team and informed by previous studies [42, 43]. Participants were asked to rank the individual program components on a 5-point Likert scale from strongly agree (=5) to strongly disagree (=1), for attractiveness (“visually appealing”), comprehension (“provided me with useful information”), usability (“easy to use/receive”), ability to persuade/engage (“helped me attain my goals”) and ability to provide support (“was supportive in answering my queries/questions”). Participants also ranked satisfaction with the overall program, individual components and length of program on a 5-point Likert scale from very satisfied (=5) to very unsatisfied (=1).

#### Estimation of treatment effect

For the primary health outcomes; physical activity level was measured via seven days of pedometry with Yamax digiwalker SW200 pedometers (Yamax Digi-Walker SW200, Kunamoto City, Japan). Diet quality was assessed using the Australian Eating Survey FFQ. From this the Australian Recommended Food Score (ARFS) diet quality index was derived using a subset of 70 items from the full FFQ. ARFS focuses on diet variety within food groups and reflects alignment with the Australian Dietary Guidelines [37], this measure has shown favourable validity and reproducibility in Australian adults [44]. Subjective well-being was determined using the Satisfaction with Life Scale (SWLS) [45], this measure has demonstrated reasonable reliability and validity among healthy young adults [46].

For the secondary health outcomes; weight, fat mass and skeletal muscle mass were measured without shoes and in light clothing using bioelectrical impedance analysis (model 720; Inbody). Height was measured to 0.1 cm on a portable stadiometer (model BSM370; InBody, Cerritos, CA). Body mass index (BMI) was calculated using height and weight data. Waist circumference was measured to 0.1 cm using a non-extensible steel tape measure. Energy intake (kJ/day), serves of fruits and vegetables and proportion of energy from alcohol, and energy-dense, nutrient poor (ED-NP) foods were measured using the validated Australian Eating Survey FFQ [47]. Self-reported moderate to vigorous physical activity (MVPA minutes/week) was assessed using the Godin Leisure-Time Exercise Questionnaire [48]. Fasting Total cholesterol, HDL-Cholesterol, LDL-Cholesterol and Triglycerides (composite measures) were measured via finger prick blood sample and analysed using the handheld CardioChek® device (Polymer Technology Systems, Inc., Indiana, US; BHR Pharmaceuticals Ltd., Nuneaton, UK). Systolic and diastolic blood pressure (composite measures), resting heart rate and augmentation index were measured using an automatic sphygmomanometer (Pulsecor Cardioscope II, Pulsecor Ltd., Auckland, New Zealand) under standardised procedures. Participants were seated for five minutes before the first blood pressure measurement and a rest period of two minutes between measures was used. Blood pressure was measured three times. An additional two measurements were taken if the blood pressure or resting heart rate values fell outside of the acceptable ranges (i.e. systolic within 10 mmHg, diastolic within 10 mmHg and resting heart rate within 5 bpm), with the mean of the two most consistent measures used. The AUDIT-C 3-item alcohol screen was used to identify hazardous drinking [49] and salivary cortisol was measured as a biomarker for psychological stress using the passive drool technique (Salimetrics LLC, SalivaBio, State College, PA 16803 USA). Self-reported measures of mental health and well-being included the Kessler psychological distress scale (K-10) [41], the Depression Anxiety Stress Scale (DASS-21) [50] the Mental Health Continuum-Short Form (MHC-SF) [51]. and the Quality of Life, Enjoyment & Satisfaction Questionnaire (Q-LES-Q) [52]. Participant demographics (age, country of birth, employment status, educational attainment, marital status and income) were recorded by questionnaire at baseline only.

### Sample size

A key objective of pilot studies is to gain initial estimates for a sample size calculation in a future adequately powered RCT [53] and thus a formal sample size calculation was not performed. A systematic review of pilot and feasibility studies identified a median total sample size of 30.5 in non-drug trials [54]. Therefore, we aimed to exceed this and a recruitment target of 50 was set.

### Randomisation

Participants were randomised by an independent research assistant who had no contact with participants during the trial. The allocation sequence was generated by a computer based random number algorithm (https://www.sealedenvelope.com/simple-randomiser/v1/lists) producing individual group allocation in block lengths of six. Randomisation codes were stored in a restricted computer folder, which was not accessible by those assessing participants or those participating in data entry for the study. Complete separation was achieved between the research assistant who generated the randomisation sequence, those who concealed allocation and from those involved in implementation of assignments.

### Statistical analysis

Data was analysed using Stata Version 12 (StataCorp. 2011. Stata Statistical Software: College Station, TX: StataCorp LP). Differences between groups at baseline were tested using independent t tests for continuous variables and chi-squared (*χ* ^2^) tests for categorical variables. The significance level for the comparison of baseline characteristics was set at 0.05. Program acceptability and satisfaction measures are presented as mean ± SD, with higher scores (maximum of 5) indicating greater acceptability/satisfaction.

For estimation of treatment effect, differences in outcomes from baseline to 3 months were tested using generalized linear mixed models for intention-to-treat (ITT) populations. Differences of means and 95% confidence intervals (CIs) were determined using the mixed models. All health outcomes were included in the model, the predictors included time (treated as categorical with levels baseline and 3 months), treatment group (intervention and control), and an interaction term for time by treatment group. Models were adjusted for baseline values of BMI, pedometer steps and proportion of energy from energy-dense, nutrient-poor foods. The *P* value associated with the interaction term was used to determine the statistical significance of any difference between treatment groups. Effect sizes were calculated using the equation: Cohen’s *d* = (M_1__change score_ – M_2 change score_)/SD_pooled [change scores]_ [55].

## Results

### Participant flow at each stage

**Fig. 1:**
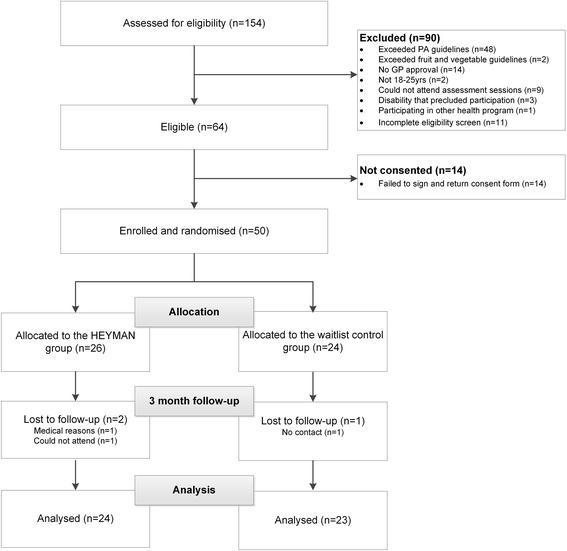
CONSORT flow chart describing the progress of participants through the trial. Flow of participants through the 3-month ‘HEYMAN’ healthy lifestyle pilot randomised controlled trial

Of the 154 young men assessed for eligibility, 64 were deemed eligible, of whom 50 were enrolled into HEYMAN and randomised into the intervention or waitlist control groups (Fig. 1).

### Baseline data

Baseline data for those randomised are summarized in Table 2. Participants had a mean age of 22.1 (SD 2.0) years, with the majority born in Australia (80%, *n* = 40). Participants were predominantly single (80.0%, *n* = 40), studying at university (62.0%, *n* = 31), in a lower income bracket earning $0 to $299 per week (48.0%, *n* = 24) and almost all (98.0% *n* = 49) had completed a high school education or higher. At baseline, participants had a mean step count of 6994.4 (SD 2421.8) steps/day and reported an average diet quality score of 29.4 (SD 9.9) out of a maximum of 73 points. The mean score for subjective well-being on the satisfaction with life scale was 23.2 (SD 6.9) out of a maximum of 35. There were no between group differences for any of the baseline demographic characteristics. There was a significant difference between groups for steps/day at baseline, with the intervention group reporting significantly more steps per day (*P* < 0.05).

**Table 2:** Baseline characteristics of the HEYMAN intervention group and the waitlist control group

Variable	Intervention group (*n* = 26)	Waitlist control group (*n* = 24)	Total (*n* = 50)
	Mean (SD) or % (*n*)		
Age	22.4 (2.0)	21.9 (2.1)	22.1 (2.0)
Country of birth:			
Australia	76.9% (20)	83.3% (20)	80.0% (40)
Other	23.1% (6)	16.7% (4)	20.0% (10)
Marital status			
Single/Divorced	88.5% (23)	79.2% (19)	84.0% (42)
Married/De facto	11.5% (3)	20.8% (5)	16.0% (8)
Employment status			
Student (University)	65.4% (17)	58.3% (14)	62.0% (31)
Student (Technical college)	3.8% (1)	0% (0)	2.0% (1)
Employed	23.1% (6)	37.5% (9)	30.0% (15)
Unemployed	7.7% (2)	4.2% (1)	6.0% (3)
Highest education level			
No formal qualification	0% (0)	4.2% (1)	2.0% (1)
Higher School Certificate	53.8% (14)	66.7% (16)	60.0% (30)
Trade/Apprenticeship	3.8% (1)	4.2% (1)	4.0% (2)
Certificate/Diploma	3.8% (1)	8.3% (2)	6.0% (3)
University degree or higher	38.5% (10)	16.7% (4)	28.0% (14)
Individual income ($AU)			
Lower ($0-$299 per week)	50.0% (13)	45.8% (11)	48.0% (24)
Middle ($300-$999 per week)	38.5% (10)	45.8% (11)	42.0% (21)
Higher ($1,000 or more per week)	11.5% (3)	4.2% (1)	8.0% (4)
Did not want to answer	0% (0)	4.2% (1)	2.0% (1)
Physical activity			
Pedometer (means steps/day)	7722.7 (2514.7)	6171.1 (2067.6)*	6994.4 (2421.8)
MVPA (minutes/week)^a^	137.2 (157.5)	108.8 (124.9)	123.3 (20.2)
Diet			
Diet quality (ARFS total score)	30.7 (8.6)	27.9 (11.2)	29.4 (9.9)
Energy intake (kJ/day)	11090.6 (3413.4)	9982.6 (3168.9)	10558.8 (3312.2)
Fruit (serves/day)	1.5 (1.2)	1.3 (1.1)	1.4 (1.2)
Vegetables (serves/day)	3.5 (1.8)	3.5 (2.6)	3.5 (2.2)
Proportion of energy from ED-NP foods (%)	40.2 (11.4)	41.8 (11.9)	41.0 (11.6)
Proportion of energy from alcohol (%)	1.7 (1.6)	2.6 (3.0)	2.1 (2.4)
Psychological measures & well-being			
Satisfaction with life scale (total score)	23.8 (6.6)	22.5 (7.4)	23.2 (6.9)
Salivary cortisol (nmol/l)	6.8 (4.8)	8.3 (4.8)	7.5 (4.8)
Depression, Anxiety & Stress Scale (total score)	11.4 (8.1)	14.1 (11.1)	12.7 (9.7)
K10 Psychological distress scale (total score)	18.5 (6.2)	21.0 (7.2)	19.7 (6.7)
Mental Health continuum- short form (total score)	44.5 (13.2)	43.8 (10.2)	44.1 (11.7)
Quality of life, enjoyment & satisfaction (total score)	49.2 (9.3)	49.3 (6.3)	49.2 (7.9)
Alcohol (AUDIT -C)			
Hazardous drinking	34.6% (9)	54.2% (13)	44.0% (22)
Weight status and body composition			
Current weight (kg)	83.6 (16.9)	80.9 (15.3)	82.3 (16.1)
Current height (cm)	179.0 (6.6)	180.3 (6.5)	179.6 (6.5)
Waist circumference	89.1 (12.0)	85.9 (11.9)	87.6 (12.0)
BMI (kg/m^2^)	26.1 (5.0)	24.8 (4.1)	25.5 (4.6)
Skeletal muscle mass (kg)	36.2 (5.8)	36.5 (4.6)	36.3 (5.2)
Body fat mass (kg)	19.9 (10.0)	16.9 (9.6)	18.5 (9.8)
BMI category (kg/m^2^)			
Healthy weight	46.2% (12)	62.5% (15)	54.0% (27)
Overweight	30.8% (8)	29.2% (7)	30.0% (15)
Obese	23.1% (6)	8.3% (2)	16.0% (8)
Cholesterol (mmol/L)			
Total cholesterol	3.9 (0.8)	3.9 (0.7)	3.9 (0.7)
HDL-cholesterol	1.2 (0.2)	1.3 (0.3)	1.3 (0.2)
LDL- cholesterol	2.2 (0.6)	2.0 (0.6)	2.1 (0.6)
Triglyceride	1.2 (0.6)	1.2 (0.7)	1.2 (0.6)
Total cholesterol/HDL-C ratio	3.3 (0.9)	3.2 (1.1)	3.2 (1.0)
Blood pressure (mmHg)			
Systolic blood pressure	120.1 (9.0)	121.6 (8.3)	120.8 (8.7)
Diastolic blood pressure	75.1 (4.7)	77.2 (5.6)	76.1 (5.2)
Resting heart rate (bpm)	69.7 (10.6)	73.8 (8.9)	71.6 (9.9)
Augmentation index (%)	40.3 (18.3)	34.6 (12.6)	37.6 (15.9)

*SD* standard deviation; Significant differences between HEYMAN group and control assessed by *t*-test or chi-square analysis

*ARFS* Australian Recommended Food Score, *ED-NP* Energy-Dense, Nutrient poor, *HDL* High Density Lipoprotein, *LDL* Low Density Lipoprotein, *MVPA* Moderate to vigorous physical activity

*p < 0.05

^a^one intervention participant removed as outlier as self- reported 7200 mins of MVPA per week

### Feasibility of research procedures

Recruitment spanned seven weeks (9^th^ March 2016 – 27^th^ April 2016) to achieve the recruitment target of 50 young men. Sharing the flyer via Facebook was the most successful recruitment method with 34% (*n* = 17) of included participants recruited this way. The second most successful recruitment strategy was flyers distributed around the University of Newcastle (20% *n* = 10), followed by recommendation from a friend (16% *n* = 8), and contact from the research team via email based on their reported interest in previous research (16% *n* = 8). Less effective recruitment strategies included; advertisements in the local newspaper (10%, *n* = 5), flyers distributed around the technical college campuses (2%, *n* = 1) and promotion of the study on a local radio station (2%, *n* = 1). Most participants who were screened and excluded were already exceeding PA guidelines (48/90).

Program retention is shown in the CONSORT flow diagram (Fig. 1). After the 3-month program final retention of participants was 94% (47/50). Although, 96% (48/50) of participants attended the post-intervention assessment session, one intervention participant started anti-psychotic medication with hyperphagic side-effects during the program which resulted in severe weight gain, elevated blood pressure and plasma cholesterol levels. Study personnel were not made aware of this until after follow-up data collection and therefore this participant was excluded from all outcome and process analysis. An additional table (Additional file 2) has been added with this participant included in analysis in order to demonstrate the impact of the medication on the individual and the impact of this on the effected outcomes. Two other young men were lost to follow-up (*n* = 1 intervention participant and *n* = 1 control participant); research assistants were unable to establish contact with one and one had moved away.

Overall, intervention participants were satisfied with their group at the time of allocation (mean ranking of 4.5 SD 0.7) and remained satisfied at the end of the program (mean ranking of 4.5 SD 0.7). Control participants were less satisfied with their allocation at both the time of allocation (mean ranking of 3.6 SD 1.0) and at program end (mean ranking of 3.7 SD 0.8). In total, 98% (49/50) of participants completed all data collection measures at baseline; one control participant failed to complete and return the seven-day pedometer record. At 3 months 100% (47/47) of those returning completed all data collection measures.

### Feasibility of implementing HEYMAN

#### Program usage

1. *Website:* Data from the process evaluation questionnaire showed that all intervention participants (100%, *n* = 24) reported visiting the website, and 62.5% (*n* = 15) reported meeting the recommended frequency of use (weekly). This was supported by data from Google Analytics™ which indicated that participants visited the website a total of 544 times, with an average of 2.10 pages/tabs viewed during each session and an average duration of one minute and 42 s. There were five featured videos on the website (four cooking videos, one exercise demonstration using the Gymstick™) which were linked to YouTube™. There were a total of 37 views across all videos with an average view duration of two minutes 40 s (average total video duration across five videos was 3 min 25 s). The ‘introduction to the Gymstick™ video’ was most watched (total 25 views) with an average view duration of 3 min 11 s (43.1% of total video duration). Next was the homemade pizza cooking video (4 views, average view duration of 1 min 25 s, 54.3% of total video duration).
2. *Jawbone*™ *wearable physical activity tracker and UP app:* Data from the process evaluation questionnaire showed that most participants (95.8%, *n* = 23) reported using the Jawbone™ and UP app, and 58.3% (*n* = 14) reported meeting the recommended frequency of use (daily). Objective data from the Jawbone UP app was available for 21 of the 24 retained participants (log in details had been changed for three participants, so sign in was not possible to access data). Additionally, an error occurred within Jawbone, which meant that no data was recorded for the final 19 days of the intervention, hence data was only available for 65 out of the 84 days. Objective data for the 21 participants indicates that all of these participants used the Jawbone UP during the intervention. Step counts were uploaded for an average of 48 (SD 19) out of the available 65 days (range of 10–65 days/participant).
3. *One-hour weekly face to face sessions:* Average attendance over the 11 group-based face-to-face sessions was 31.3% (*n* = 7.5) and 8.3% (*n* = 2) met the recommended attendance rates (weekly). Most participants (95.8%, *n* = 23) attended the one-to-one individualised session in person. One remaining participant attended via telephone. Although 91.7% (*n* = 22) were identified as meeting the recommended attendance frequency for the one-to-one individualised session (one 60-min session), an additional participant reported not attending this session in the process evaluation survey, despite objective attendance records showing his presence.
4. *Personalised food and nutrient report:* All participants (100%, *n* = 24) completed the Australian Eating Survey FFQ at baseline and received the personalised food and nutrient intake report during the one-to-one individualised session. The one participant who could not attend the one-to-one individualised session in-person but attended via telephone was send the report via email.
5. *A private Facebook discussion group:* All participants (100%, *n* = 24) joined the program Facebook group, with a total of 23 posts, including 22 posts by the moderator. There was an average of 20 views and 1.8 ‘likes’ per post. In total, 75% (*n* = 18) reported meeting the recommended frequency of use (reading weekly Facebook posts).
6. *Gymstick*™ *resistance band:* Most (95.8%, *n* = 23) reported using the Gymstick™ resistance training equipment and 33.3% (*n* = 8) met the recommended frequency of use (twice weekly).
7. *TEMPlate*™ *dinner disc:* Overall, 66.7% (*n* = 16) reported using the TEMPlate™ dinner disc, but none met the recommended frequency of use (daily).

#### Acceptability of program components (attractiveness, comprehension, usability, supportiveness, satisfaction and ability to persuade)

Table 3 summarizes the mean rankings for program acceptability. Responses indicate participants found all program components easy to understand (mean scores, 4.1–4.4) and most program components easy to use/navigate (mean scores, 3.5-4.3), with the website reported as being the easiest to use (mean, 4.3 SD 0.6). Most program components were found to be visually appealing (mean scores, 3.4–4.0). The individualised one-to-one session was ranked highest for providing useful information about healthy eating (mean, 4.5 SD 0.7), physical activity (mean, 4.2 SD 0.8) and stress (mean, 4.0 SD 0.9). All program components were ranked favourably (mean scores, 3.3–4.1) for helping participants attain their goals, with the personalised food and nutrient report ranked highest with a mean score of 4.1 (SD 1.0). Most program components motivated participants (mean scores, 3.3–4.3) and made them feel accountable (mean scores, 3.2–4.2). Furthermore, participants felt that the face-to-face sessions and Facebook group were supportive in answering any queries/questions (mean scores, 3.7–4.5).

**Table 3:** Rankings for attractiveness, comprehension, usability, supportiveness, satisfaction and ability to persuade for program components^a^

	Website (*n* = 24)	Jawbone™/UP app (*n* = 23)	F2F (group) (*n* = 19)	F2F (1-2-1) (*n* = 22)	Facebook group (*n* = 24)	Food & nutrient report (*n* = 24)	Gymstick™ (*n* = 23)	TEMPlate™ dinner disc (*n* = 16)
Provided me with useful information about healthy eating	4.0 ± 0.6	3.2 ± 1.0	4.1 ± 0.6	4.5 ± 0.7	3.8 ± 0.8	4.4 ± 0.9	NA	3.9 ± 0.9
Provided me with useful information about exercise	4.2 ± 0.6	3.9 ± 0.9	4.2 ± 0.9	4.2 ± 0.8	3.7 ± 0.8	NA	NA	NA
Provided me with useful information about stress	3.7 ± 0.7	3.0 ± 0.9	3.6 ± 0.8	4.0 ± 0.9	3.5 ± 0.8	NA	NA	NA
Helped me to attain my goals	3.6 ± 0.8	3.8 ± 0.9	3.9 ± 1.0	4.0 ± 0.9	3.7 ± 0.9	4.1 ± 1.0	3.7 ± 1.0	3.3 ± 0.9
Motivated me	3.5 ± 0.9	4.1 ± 0.7	4.3 ± 0.7	4.0 ± 0.8	3.6 ± 0.8	4.0 ± 0.9	3.5 ± 1.0	3.3 ± 1.1
Made me feel accountable	3.3 ± 0.9	4.0 ± 1.0	3.8 ± 0.9	4.1 ± 0.8	3.5 ± 1.0	4.2 ± 0.9	3.6 ± 1.0	3.2 ± 0.9
Was easy to use/navigate	4.3 ± 0.6	4.0 ± 0.9	NA	NA	4.2 ± 0.8	NA	3.8 ± 1.0	3.5 ± 1.2
Content was easy to understand	4.3 ± 0.4	4.1 ± 0.7	4.4 ± 0.6	4.4 ± 0.6	4.2 ± 0.8	4.1 ± 0.9	NA	NA
Was visually appealing	4.0 ± 0.7	4.0 ± 0.9	NA	NA	3.8 ± 0.8	3.9 ± 0.9	NA	3.4 ± 0.9
Was supportive in answering any queries/questions	NA	NA	4.5 ± 0.7	4.5 ± 0.6	3.7 ± 0.8	NA	NA	NA
Satisfaction	4.0 ± 0.6	4.2 ± 1.1	4.1 ± 0.8	4.3 ± 0.8	Not asked	4.2 ± 0.8	4.0 ± 0.9	3.0 ± 0.9

*F2F* Face-to-face, *NA* Not applicable

Data are mean ± standard deviation values

^a^Maximum score = 5

Overall, 87.5% (*n* = 21) of participants reported they were very satisfied or satisfied with the program, 12.5% (*n* = 3) were neutral and no participant reported being unsatisfied or very unsatisfied. Of all program components, participants were most satisfied with the one-to-one individualised session (mean, 4.3 SD 0.8), the Jawbone™ fitness band/UP app (mean, 4.2, SD 1.1) and the personalised food and nutrient report (mean, 4.2, SD 0.8), and least satisfied with the TEMPlate™ dinner disc (mean, 3.0 SD 0.9). In addition, participants found that the 12-week intervention period was long enough (mean, 4.0 SD 0.8).

### Estimation of treatment effect

Table 4 summarises the results of the intention-to-treat analysis examining baseline to 3-month differences between the intervention and control groups for all outcomes.

**Table 4:** Mean change in outcomes within groups and differences between groups (Intention-to-Treat Populations) at 3 months

	Mean change from baseline (95%CI)^a^				
Outcomes^c^	Control group (*n* = 24)	Intervention group (*n* = 26)	Mean difference between groups (95%CI)^b^	*p*-Value	Effect size (Cohen’s *d*)
Physical activity (pedometer steps/day)	575.4 (−518.8, 1669.7)	1588.2 (534.7, 2641.6)	1012.7 (−506.2, 2531.6)	0.191	0.36
Diet quality (ARFS total score)	2.3 (−0.5, 5.2)	5.9 (3.1, 8.7)	3.6 (−0.4, 7.6)	0.081	0.48
Satisfaction with life scale (total score)	0.5 (−0.9, 2.0)	0.9 (−0.5, 2.4)	0.4 (−1.6, 2.5)	0.683	0.11
Fruit (serves/day)	0.3 (−0.1, 0.7)	0.5 (0.1, 0.9)	0.2 (−0.4, 0.8)	0.496	0.20
Vegetables (serves/day)	−0.1 (−0.8, 0.6)	1.0 (0.3, 1.6)	1.1 (0.1, 2.0)	*<0.05*	0.62
Energy intake (kJ/day)	−68.9 (−1090.7, 953.0)	−475.9 (−1470.6, 518.8)	−407.0 (−1833.1, 1019.0)	0.576	0.16
Proportion of energy from ED-NP foods (%)	−2.6 (−6.2, 1.1)	−9.8 (−13.3, −6.2)	−7.2 (−12.3, −2.1)	*<0.01*	0.73
Proportion of energy from alcohol (%)	−0.5 (−1.2, 0.3)	0.2 (−0.5, 1.0)	0.7 (−0.3, 1.8)	0.181	0.36
AUDIT-C (total score)	−0.6 (−1.1, −0.1)	0.1 (−0.4, 0.6)	0.7 (−0.1, 1.4)	0.074	0.53
Salivary Cortisol (nmol/l)	2.7 (−0.4, 5.8)	0.6 (−2.4, 3.6)	−2.1 (−6.4, 2.2)	0.341	0.27
Depression, Anxiety & Stress Scale (total score)	−2.1 (−4.8, 0.5)	−1.7 (−4.3, 0.9)	0.4 (−3.3, 4.1)	0.835	0.06
K-10 Psychological distress scale	−1.7 (−3.0, −0.4)	−2.6 (−3.8, −1.3)	−0.9 (−2.6, 0.9)	0.331	0.28
Mental Health Continuum (total score)	4.0 (0.8, 7.1)	5.5 (2.4, 8.6)	1.5 (−2.9, 6.0)	0.505	0.19
Quality of Life, Enjoyment and Satisfaction (total score)	2.9 (0.1, 5.6)	6.0 (3.3, 8.7)	3.2 (−0.7, 7.0)	0.110	0.45
Weight (kg)	1.0 (0.1, 2.0)	−0.6 (−1.5, 0.3)	−1.6 (−3.0, −0.3)	*<0.05*	0.63
Weight (%)	1.3 (0.2, 2.5)	−0.6 (−1.7, 0.5)	−2.0 (−3.5, −0.4)	*<0.05*	0.67
Waist circumference (cm)	1.9 (0.7, 3.1)	−1.2 (−2.4, −0.0)	−3.1 (−4.8, −1.4)	*<0.001*	0.89
BMI (Kg/m^2^)	0.3 (0.0, 0.6)	−0.2 (−0.5, 0.0)	−0.6 (−0.9, −0.2)	*<0.01*	0.81
Skeletal muscle mass (kg)	0.1 (−0.5, 0.6)	−0.0 (−0.6, 0.5)	−0.1 (−0.9, 0.6)	0.755	0.07
Body fat mass (kg)	0.9 (0.1, 1.7)	−0.5 (−1.3, 0.3)	−1.4 (−2.5, −0.3)	*<0.05*	0.67
MVPA (minutes/week)	26.1 (−58.0, 110.2)	154.1 (71.0, 237.1)	128.0 (9.8, 246.2)	*<0.05*	0.58
Total cholesterol (mmol/l)	0.0 (−0.2, 0.3)	−0.4 (−0.6, −0.2)	−0.4 (−0.8, −0.1)	*<0.05*	0.61
HDL-Cholesterol (mmol/l)	0.0 (−0.0, 0.1)	0.0 (−0.0, 0.1)	0.0 (−0.1, 0.1)	0.986	0.00
LDL- Cholesterol (mmol/l)	0.1 (−0.1, 0.3)	−0.4 (−0.6, −0.1)	−0.5 (−0.8, −0.2)	*<0.01*	0.83
Triglyceride (mmol/l)	−0.2 (−0.5, −0.0)	−0.0 (−0.3, 0.2)	0.2 (−0.1, 0.6)	0.200	0.31
Total cholesterol/HDL-C ratio	−0.0 (−0.2, 0.2)	−0.3 (−0.5, −0.2)	−0.3 (−0.6, −0.0)	*<0.05*	0.60
Systolic blood pressure (mm Hg)	−2.6 (−5.5, 0.2)	−2.4 (−5.1, 0.4)	0.3 (−3.7, 4.2)	0.898	0.04
Diastolic blood pressure (mm Hg)	−1.1 (−2.9, 0.7)	−1.7 (−3.5, 0.0)	−0.6 (−3.1, 1.9)	0.629	0.13
Resting Heart rate (BPM)	−2.1 (−6.1, 1.9)	−0.1 (−4.0, 3.8)	2.0 (−3.6, 7.6)	0.480	0.20
Augmentation Index (%)	−0.7 (−8.1, 6.6)	−4.8 (−11.9, 2.3)	−4.1 (−14.3, 6.2)	0.438	0.22

*ARFS* Australian Recommended Food Score, *ED-NP* Energy-Dense, Nutrient poor, *HDL* High Density Lipoprotein, *LDL* Low Density Lipoprotein, *MVPA* Moderate to vigorous physical activity

^a^Time differences were calculated as (3 months – baseline)

^b^Between group differences at 3 months

^c^Adjusted for baseline values of BMI, physical activity steps and proportion of energy from Energy-dense, Nutrient poor (ED-NP) foods

#### Changes in pedometer steps/day, diet quality and well-being (primary outcomes for subsequent RCT)

No significant differences between groups were observed for pedometer steps/day (1012.7 steps/day, 95% CI = −506.2, 2531.6, *p* = 0.191, Cohen’s *d* = 0.36), diet quality score (3.6, 95% CI = −0.4, 7.6, *p* = 0.081, *d* = 0.48) or total well-being score from the satisfaction with life scale (0.4, 95% CI = −1.6, 2.5, *p* = 0.683, *d* = 0.11). Significant within group differences were evident in the intervention group for pedometer steps/day (1588.2 steps/day, 95% CI = 534.7, 2641.6) and diet quality score (5.9 95% CI = 3.1, 8.7).

#### Changes in lifestyle, psychological, anthropometric and physiological measures (secondary outcomes in the subsequent RCT)

Significant differences favouring the intervention group over the control group at 3-months were observed for self-reported MVPA (*p* < 0.05, *d* = 0.58), daily vegetable servings (*p* < 0.05, *d* = 0.62), percentage energy from ED-NP foods (*p* < 0.01, *d* = 0.73), weight (*p* < 0.05, *d* = 0.63), percentage weight loss (*p* < 0.05, *d* = 0.67), waist circumference (*p* < 0.001, *d* = 0.89), BMI (*p* < 0.01, *d* = 0.81), body fat mass (*p* <0.05, *d* = 0.67), plasma total cholesterol (*p* < 0.05, *d* = 0.60), LDL cholesterol (*p* < 0.01, *d* = 0.83) and ratio of total cholesterol-to-HDL cholesterol (*p* < 0.05, *d* = 0.60).

## Discussion

This pilot RCT tested the feasibility of the ‘HEYMAN’ healthy lifestyle program for young adult men (aged 18–25 years). The research procedures (recruitment, randomisation, data collection and retention) and intervention components (program usage, attractiveness, comprehension, usability, support, satisfaction and ability to persuade) were generally feasible and acceptable. In addition, the current study aimed to estimate the HEYMAN treatment effects on improving pedometer steps/day, diet quality and well-being (*primary outcomes*) and a number of lifestyle, psychological, anthropometric and physiological measures (*secondary outcomes*). Overall, results indicated that the research procedures and study protocol were adequate to proceed to a full scale RCT, with some modifications required. The HEYMAN program also demonstrated potential for positively impacting on a number of health outcomes.

### Feasibility

Challenges associated with recruiting young men have been widely acknowledged and explain the scarcity of lifestyle behaviour interventions in this population group [24]. The current study was successful in recruiting 50 young men over a seven-week period. The current study demonstrated high retention rates with 94% (47/50) returning at the 3-month follow-up, which is particularly promising given previous interventions have demonstrated difficulties in retaining young men. A recent systematic review highlighted that only three of 10 health-behaviour interventions in young men met the criteria for adequate retention (defined as a dropout of ≤20% for ≤6-month follow-up or ≤30% for > 6-month follow-up) [24]. However, a significant strength of the HEYMAN program was development based on guidance from a participatory research model (PRECEDE-PROCEED), where formative research with young men informed program development, design, messaging and delivery. Thus recruitment materials and program messages were focussed around young men’s individual preferences and addressed perceived motivators and barriers (e.g., focusing on fitness and strength). Also branding and graphic design throughout the program materials reflected young men (e.g., images of young men) and other male engagement strategies were used (e.g. sensitive use of humour) [56]. This is likely to have increased the appeal of the program and ultimately enabled successful recruitment and retention. One issue requiring modification for the full scale RCT was with the exclusion criteria, which meant a large proportion of young men who required assistance with improving health behaviours could not take part. Particularly, many were identified as having poor eating habits but exceeded national recommendations for physical activity guidelines and thus were excluded. Adapting the eligibility criteria to include young men who fail to meet national recommendations for *either* dietary behaviours *or* physical activity rather than both, could address current challenges in recruiting larger numbers of young men in total and in a shorter time period.

Randomisation to start HEYMAN immediately or after a 3-month wait was shown to be feasible and generally acceptable to participants. As expected, controls reported to be less satisfied with allocation but there were no differences in drop-out at 3 months and almost all of controls were retained (23/24). To maximise retention of control participants, participants were informed of incentives for returning to the follow-up session, including a voucher to cover travel expenses and free exercise equipment at program commencement. Future research with young men could adopt similar techniques to prevent attrition.

Within a single assessment session, the number of instruments used to measure outcomes should be minimised to prevent excessive participant burden [57]. The considerable number of outcomes measured within the HEYMAN pilot was intensive. Despite this, retention through the trial remained high. The strategy of providing participants with self-report measures to be completed at home was effective and helped to minimise participant burden.

Young men reported the intervention was acceptable and were generally satisfied with most program components. A strength of the HEYMAN program was the use of socio-culturally relevant content and strategies when presenting and delivering intervention messages to young men [22]. In further support of this, 83% of participants selected either ‘agree’ or ‘strongly agree’ for the appropriateness of the HEYMAN program because it was clearly designed for young men. Also the use of behaviour change theories and responses from formative work with young men to inform overall development further strengthened the program. The individualised face-to-face session ranked highest (or joint highest) for support, attaining goals, comprehension and satisfaction. This was to be anticipated since the benefits of this method have been illustrated in other interventions. For example, a review of 10 studies indicated that the most effective physical activity interventions were those that offered both individual and group support and using a tailored approach [58]. Similarly, a review of 21 studies established the benefits of individual dietary counselling on reducing saturated fats and increasing intake of fruit and vegetables [59].

Young men demonstrated reasonable compliance and acceptability with most of the eHealth components of the program including the website and Jawbone™ wearable device, and associated UP app. This was expected given the high level of engagement with technology in this population group [60]. There were a total of 544 website views during the 3-month intervention period and process evaluation data revealed all participants had used the website. A comparable healthy lifestyle intervention in young adults (including 39% young men) demonstrated lower engagement, with less than half (43%) of participants reported to have used the website [61]. Declining website usage as the intervention progresses is common [62]. To maintain engagement with the HEYMAN website, new online content was introduced regularly throughout the program, which appeared to be effective as website views remained consistent, with views in week one (90 website visits) comparable to week seven (77 website visits) and week 11 (75 website visits). Despite frequent visits to the HEYMAN website, the average duration was short (one minute and 42 s). This may be due to the brief content on some pages that was linked out to other resources, so time spent on each page may not be a true reflection of engagement. Total views of the featured website videos were low, which suggests that alternative ways of relaying this information (i.e., during the individualised sessions) may be more acceptable.

Objective data for the Jawbone UP was available for 21 participants and all had used this during the intervention. On average, step counts were uploaded for 48 out of the available 65 days, equating to around 74% of the available time period. Usage in this current study appears to be slightly higher when compared to a similar study with middle-aged Australian men which identified 59% of the sample to have used the Jawbone UP during the 20-week intervention, with step counts uploaded for 69% of total time period (average of 96 out of 140 days) [63]. A substantial expense was providing all individuals with the Jawbone™ wearable physical activity tracker. The financial burden associated with this may affect the scalability of the full scale HEYMAN with a large sample. If sufficient funds are not available, some modifications could be applied to the full scale RCT including; replacing the Jawbone™ component with a smartphone device as many young adults already use a smartphone [64] and many of these have built-in step-trackers. Alternatively, the program could employ a ‘bring your own device’ plan for those who already use wearable devices. We are unaware if participants will demonstrate corresponding satisfaction and acceptability scores with these alternative options. However, these options will still enable goal-setting and self-monitoring of physical activity on a lower budget.

Even though all participants joined the Facebook group and engaged with the content (average of 20 views per post and 75% reported reading the weekly posts), the compliance and acceptability was much lower for the Facebook group as a social support tool. Only one participant posted to the Facebook discussion group with the remaining posts by the moderator. This is consistent with a similar targeted program for men which reported engagement with social forums/discussion groups as low [65]. However a recent weight loss program with young adults (including 29.7% young men) found Facebook to be the primary modality for delivery of dynamic content at the group level [66]. The context of Facebook was utilised as a social support tool via a number of challenges and targeted campaigns. For example; facilitators challenged participants to not eat candy for two weeks. Participants publicly accepted the challenge by posting on Facebook for their friends to see. Participants then posted methods used to meet the goal and the facilitator provided feedback on methods, encouraged self-monitoring, and prompted goal review. Subsequently, the facilitator and each participant’s social network provided social support and accountability through posts, comments, or likes until the campaign ended. Therefore, Facebook may not have been used to its full potential in HEYMAN by both intervention deliverers and participants. As a result the low acceptability may not be due to the technology itself, but due to the method of presenting the content. Therefore future versions of HEYMAN could consider alternative methods of social support through Facebook.

In moving forward, measures of technology usage should not be seen as the only determinant of engagement [67]. If a participant used/visits an eHealth component, it does not mean they are engaged by it. Therefore, multiple measures of engagement, including iterative, in-depth mixed methods research is required to fully understand and address issues affecting user engagement and to fully determine what constitutes ‘effective engagement’ i.e., engagement associated with positive interventions outcomes [67].

Although the weekly group face-to-face sessions ranked highly for supportiveness, motivation, comprehension and attaining goals, average attendance was low (31.3%), particularly when compared to another targeted health program for middle-aged men which reported average attendance as 86% [68]. However this study by Hooker et al. [68] only included group based face-to-face sessions as the sole delivery mode. HEYMAN included different intervention components to relay information and resources across different mediums (i.e., exercises in the group sessions could also be accessed on the website and completed at home). This approach coincided with previous formative research in young men who expressed their preference for being able to access multiple delivery mediums [36] and acknowledged the need to address key barriers to PA and healthy eating including; “busy lifestyles” and “lack of time” [24, 35]. Due to the greater variety of options, participants may have chosen different delivery modes to suit their lifestyles. Despite the low attendance, 71% of participants reported being very satisfied or satisfied with the session content. The attendance levels were sporadic throughout the program with many citing work and study commitments for non-attendance, despite young men previously expressing preference for this delivery mode [36]. The group based face-to-face sessions had a fixed time slot, but in progressing to the full scale RCT, greater flexibility may be required to enhance attendance, for example, communicating with potential participants to establish the most convenient days and times for sessions and possibly opening up alternative days and times to run the sessions. Also, given the high attendance, acceptability and satisfaction with the individualised one-to-one sessions, future versions of HEYMAN could trial more individualised sessions and fewer group based sessions. But in doing this, researcher burden will be increased and therefore delivery via video consultations using software such as Skype, Google Hangout or other programs could also be considered.

The TEMPlate™ dinner disc to guide portion sizes at meal times demonstrated low acceptability and satisfaction. Although most used the disc initially (66.7%, *n* = 16), none used it daily. Open responses citing reasons for non-use were mainly centred around the inconvenience and impracticality for many types of food. Especially for foods that could not be ‘deconstructed’ to align with the sections on the plate. As a result, this component should be removed in future trials and portion size education via the website, apps or in face-to-face sessions trialled.

### Estimation of treatment effect

Although this pilot RCT was not designed to be powered to detect between-group differences in outcomes, a number of significant results were still observed. This indicates the potential effectiveness of a future fully powered RCT. Moderate (*d* >0.50) to large (*d* > 0.80) effects were observed for changes in MVPA, vegetable serves, energy from ED-NP foods, weight, percent weight loss, waist circumference, BMI, body fat mass, total cholesterol, LDL cholesterol and total cholesterol-to-HDL cholesterol ratio. There were no significant intervention effects observed for any of the primary outcomes for pedometer steps/day, diet quality or subjective well-being. However, the medium effect size for ARFS diet quality score (*p =* 0.08, *d* = 0.48) is promising. Greater improvements were observed when compared to a study of overweight and obese adults in an online weight loss RCT, at 3-months [69]. The ARFS diet quality score focuses on variety within the core (healthy) food groups recommended in the Australian Dietary Guidelines [37]. When examining ARFS subscales in the current study (see Additional file 3), future interventions should encourage a greater variety of foods within the core food groups of wholegrains, vegetables, meat, vegetarian alternatives and dairy products. Also, despite significant reductions in ED-NP foods in the intervention group, the percentage of total energy intake from these foods was still much higher than the recommended maximum limit of 15% of total energy intake, based on consuming up to 3 servings per day [37]. Future interventions should provide a greater focus on strategies to reduce ED-NP intakes. The difference in number of steps between intervention and control (1012.7 steps per day) was less than results from a meta-analysis of eight RCT’s, which reported a between group difference of 2491 steps per day [70]. However, two participants in the HEYMAN intervention group had serious injuries at follow-up measurement, which impeded their level of activity and may have contributed to the smaller differences between groups.

There was a small effect (*d* = 0.11) on subjective well-being score from the satisfaction with life scale. Also effect sizes were small for all secondary psychological measures. Potential reasoning for this may be due to over half of the sample being University students (62%) and the follow-up measures closely followed an examination period. A previous study has indicated that studying for examinations, undertaking examinations and receiving examination results were the highest causes of psychological distress among University students [71].

For several outcomes including diet quality, pedometer steps and psychological measures, small but positive changes were observed by the control group. For that reason, the overall treatment effect may have been underestimated. These improvements may occur in response to undergoing baseline assessment, due to participants’ awareness of being involved in an experimental trial, or anticipation of receiving active treatment [72]. Also most positive changes for controls were observed in outcomes with self-report measures, which are subject to social desirability bias [73], and therefore self-reported behaviour changes in controls may reflect unreliable reporting, rather than actual behaviour changes [72].

### Limitations

Although significant between group differences were identified for a number of health outcomes at 3-months, these results are indicative rather than conclusive. Given this was a pilot trial, the sample was purposely small, with low power and prevents any conclusions being made regarding the generalisability of findings. The short duration of the trial also limits the interpretation of these findings. Future versions of the HEYMAN program could include longer follow-up to assess long terms effects. The use of self-report measures also introduces reporting bias and an error in the objective Jawbone data meant that data was only available for 65 out of the 84 days. Despite best efforts to recruit a diverse sample of young men, there was an over-representation of those with higher education levels and in full-time study. In addition, participants were de-identified for the Google Analytics data to determine website usage, therefore we could not separate data by completers and drop-outs. The assessment for retention (i.e., those completing follow-up measures) may be considered a limitation as some participants may have attended and completed follow-up measures but not completed the intervention (i.e., non-usage attrition).

## Conclusions

Young adulthood is an ideal time to intervene on health behaviours in order to prevent or delay serious mental and/or physical health problems. However, young men have been under-represented in interventions to improve health behaviours, and the few that have been carried out have experienced problems with reaching and retaining them. Participatory-based interventions such as HEYMAN, which involve young men in the program design and match program content and messages to their expressed preferences, needs and barriers, has the potential to overcome limitations previously identified. Findings from the current pilot study demonstrated sufficient feasibility in terms of research procedures, including recruitment, retention, randomisation and data collection to justify progress to a full scale RCT, with some minor amendments necessary. To improve overall acceptability, engagement and satisfaction, modifications are required including; changing the composition of face-to-face sessions with an increased number of individualised sessions and greater flexibility in the time and days for group based sessions. Some adaptations to the Facebook content is required and including portion size education across a number of delivery mediums. The lessons learnt from the current pilot study provide key insights into working with young men. Results demonstrated that HEYMAN has the potential to successfully facilitate some lifestyle changes and improve a number of health behaviours in young men.

## Additional files

Additional file 1: Full list of HEYMAN components and alignment to participatory responses and behaviour change strategies. (DOCX 24 kb)
Additional file 2: Mean change in outcomes within groups and differences between groups (Intention-to-Treat Populations) at 3 months - results with follow-up data for the withdrawn participant for outcomes effected by medication. (DOCX 14 kb)
Additional file 3: Mean change in subscales of the ARFS within groups and differences between groups (Intention-to-Treat Populations) at 3 months. (DOCX 13 kb)

## Abbreviations

BMI: Body mass index
CI: Confidence intervals
DASS-21: Depression, anxiety and stress scale
ED-NP: Energy-dense, nutrient poor
FFQ: Food frequency questionnaire
HDL: High density lipoprotein
HEYMAN: Harnessing ehealth to enhance young men’s mental health, activity and nutrition
LDL: Low density lipoprotein
MVPA: Moderate to vigorous physical activity
PA: Physical activity
RCT: Randomised controlled trial
SCT: Social cognitive theory
SDT: Self-determination theory

## References

[1] Mullen K, Watson J, Swift J, Black D (2007). Young men, masculinity and alcohol. Drugs: Education, Prevention, Policy.

[2] Poobalan AS, Aucott LS, Precious E, Crombie IK, Smith WC (2010). Weight loss interventions in young people (18 to 25 year olds): a systematic review. Obes Rev.

[3] Alcohol Alert: Young Adult Drinking [http://pubs.niaaa.nih.gov/publications/aa68/aa68.htm]. Accessed 16 June 2016.

[4] Irazusta A, Hoyos I, Irazusta J, Ruiz F, Díaz E, Gil J (2007). Increased cardiovascular risk associated with poor nutritional habits in first-year university students. Nutr Res.

[5] Li K-K, Concepcion RY, Lee H, Cardinal BJ, Ebbeck V, Woekel E, Readdy RT (2012). An examination of sex differences in relation to the eating habits and nutrient intakes of university students. J Nutr Educ Behav.

[6] Keating XD, Guan J, Piñero JC, Bridges DM (2005). A meta-analysis of college students’ physical activity behaviors. J Am Coll Heal.

[7] Lake AA, Adamson AJ, Craigie AM, Rugg-Gunn AJ, Mathers JC (2009). Tracking of dietary intake and factors associated with dietary change from early adolescence to adulthood: the ASH30 study. Obesity facts.

[8] Spring B, Moller AC, Colangelo LA, Siddique J, Roehrig M, Daviglus ML, Polak JF, Reis JP, Sidney S, Liu K. Healthy lifestyle change and subclinical atherosclerosis in young adults: Coronary Artery Risk Development in Young Adults (CARDIA) study. Circ. 2014;130(1):10–7. 10.1161/CIRCULATIONAHA.113.005445 PMC4615574 24982115

[9] Liu K, Daviglus ML, Loria CM, Colangelo LA, Spring B, Moller AC, Lloyd-Jones DM (2012). Healthy lifestyle through young adulthood and the presence of Low cardiovascular disease risk profile in middle Age the coronary artery risk development in (young) adults (CARDIA) study. Circulation.

[10] Sanchez-Villegas A, Ara I, Guillen-Grima F, Bes-Rastrollo M, Varo-Cenarruzabeitia JJ, Martinez-Gonzalez MA (2008). Physical activity, sedentary index, and mental disorders in the SUN cohort study. Med Sci Sports Exerc.

[11] Akbaraly TN, Brunner EJ, Ferrie JE, Marmot MG, Kivimaki M, Singh-Manoux A (2009). Dietary pattern and depressive symptoms in middle age. Br J Psychiatry.

[12] Neckelmann D, Mykletun A, Dahl AA (2007). Chronic insomnia as a risk factor for developing anxiety and depression. SLEEP-NEW YORK THEN WESTCHESTER-.

[13] Wilcox HC, Conner KR, Caine ED (2004). Association of alcohol and drug use disorders and completed suicide: an empirical review of cohort studies. Drug Alcohol Depend.

[14] Burns J, Davenport TA, Christensen H, Luscombe GM, Mendoza JA, Bresnan A, Blanchard ME, Hickie IB (2013). Game on: exploring the impact of technologies on young Men’s mental health and wellbeing. Findings from the first young and well national survey.

[15] Parker ED, Schmitz KH, Jacobs DR, Dengel DR, Schreiner PJ (2007). Physical activity in young adults and incident hypertension over 15 years of follow-up: the CARDIA study. Am J Public Health.

[16] Pereira MA, Kartashov AI, Ebbeling CB, Van Horn L, Slattery ML, Jacobs DR, Ludwig DS (2005). Fast-food habits, weight gain, and insulin resistance (the CARDIA study): 15-year prospective analysis. Lancet.

[17] Ashton LM, Hutchesson MJ, Rollo ME, Morgan PJ, Thompson DI, Collins CE (2015). Young adult males’ motivators and perceived barriers towards eating healthily and being active: a qualitative study. Int J Behav Nutr Phys Act.

[18] Crane MM, LaRose JG, Espeland MA, Wing RR, Tate DF (2016). Recruitment of young adults for weight gain prevention: randomized comparison of direct mail strategies. Trials.

[19] Tate DF, LaRose JG, Griffin LP, Erickson KE, Robichaud EF, Perdue L, Espeland MA, Wing RR (2014). Recruitment of young adults into a randomized controlled trial of weight gain prevention: message development, methods, and cost. Trials.

[20] Bost ML (2005). A descriptive study of barriers to enrollment in a collegiate health assessment program. J Community Health Nurs.

[21] Moe SG, Lytle LA, Nanney MS, Linde JA, Laska MN (2016). Recruiting and retaining young adults in a weight gain prevention trial: lessons learned from the CHOICES study. Clinical Trials.

[22] Morgan PJ, Young MD, Smith JJ, Lubans DR (2016). Targeted health behavior interventions promoting physical activity: a conceptual model. Exerc Sport Sci Rev.

[23] Ashton LM, Hutchesson MJ, Rollo ME, Morgan PJ, Collins CE (2014). A scoping review of risk behaviour interventions in young men. BMC Public Health.

[24] Ashton LM, Morgan PJ, Hutchesson MJ, Rollo ME, Young MD, Collins CE (2015). A systematic review of SNAPO (smoking, nutrition, alcohol, physical activity and obesity) randomized controlled trials in young adult men. Prev Med.

[25] Galani C, Schneider H (2007). Prevention and treatment of obesity with lifestyle interventions: review and meta-analysis. Int J Public Health.

[26] Bertholet N, Daeppen J-B, Wietlisbach V, Fleming M, Burnand B (2005). Reduction of alcohol consumption by brief alcohol intervention in primary care: systematic review and meta-analysis. Arch Intern Med.

[27] Oliffe JL, Greaves L (2012). Designing and conducting gender, Sex, and health research.

[28] Oakley A, Strange V, Bonell C, Allen E, Stephenson J (2006). Process evaluation in randomised controlled trials of complex interventions. BMJ (Clinical research ed).

[29] Bauman A, Nutbeam D. Evaluation in a nutshell: a practical guide to the evaluation of health promotion programs. USA, New York: McGraw Hill; 2013.

[30] Thabane L, Ma J, Chu R, Cheng J, Ismaila A, Rios LP, Robson R, Thabane M, Giangregorio L, Goldsmith CH (2010). A tutorial on pilot studies: the what, why and how. BMC Med Res Methodol.

[31] Moher D, Schulz KF, Altman DG (2001). The CONSORT statement: revised recommendations for improving the quality of reports of parallel group randomized trials. BMC Med Res Methodol.

[32] Gielen A, McDonald E, Gary T, Bone L, Glanz K, Rimer B, Viswanath K (2008). Using the PRECEDE-PROCEED model to appply health behavior theories. Health behavior and health education: theory, research, and practice.

[33] Crosby R, Noar SM (2011). What is a planning model? An introduction to PRECEDE‐PROCEED. J Public Health Dent.

[34] Viswanathan M, Ammerman A, Eng E, Garlehner G, Lohr KN, Griffith D, Rhodes S, Samuel-Hodge C, Maty S, Lux L (2004). Community‐based participatory research: assessing the evidence: summary.

[35] Ashton L, Hutchesson M, Rollo M, Morgan P, Collins C. Motivators and barriers to engaging in healthy eating and physical activity: A cross-sectional survey in young adult men. Am J Mens Health. 2016:1–14. Epub ahead of print. 10.1177/1557988316680936 PMC5675273 27923963

[36] Ashton L (2016). Development and evaluation of the HEYMAN (Harnessing Ehealth to enhance Young men’s Mental health, Activity and Nutrition) healthy lifestyle program for young adult men aged 18–25 years (unpublished doctoral thesis - Under examination).

[37] National Health and Medical Research Council (NHMRC): Eat for Health: Australian Dietary Guidelines. Canberra: Australian Government; Department of Health; 2013.

[38] Australia's Physical Activity and Sedentary Behaviour Guidelines [http://www.health.gov.au/internet/main/publishing.nsf/content/health-pubhlth-strateg-phys-act-guidelines]. Accessed 7 Nov 2015.

[39] Bandura A (1986). Social foundations of thought and action: A social cognitive theory.

[40] Ryan RM, Deci EL (2000). Self-determination theory and the facilitation of intrinsic motivation, social development, and well-being. Am Psychol.

[41] Kessler RC, Andrews G, Colpe LJ, Hiripi E, Mroczek DK, Normand S-L, Walters EE, Zaslavsky AM (2002). Short screening scales to monitor population prevalences and trends in non-specific psychological distress. Psychol Med.

[42] Morgan PJ, Scott HA, Young MD, Plotnikoff RC, Collins CE, Callister R (2014). Associations between program outcomes and adherence to social cognitive theory tasks: process evaluation of the SHED-IT community weight loss trial for men. Int J Behav Nutr Phys Act.

[43] Hutchesson MJ, Morgan PJ, Callister R, Pranata I, Skinner G, Collins CE (2016). Be positive Be health e: development and implementation of a targeted e-health weight loss program for young women. Telemedicine e-Health.

[44] Collins CE, Burrows TL, Rollo ME, Boggess MM, Watson JF, Guest M, Duncanson K, Pezdirc K, Hutchesson MJ (2015). The comparative validity and reproducibility of a diet quality index for adults: the Australian recommended food score. Nutrients.

[45] Diener E, Emmons RA, Larsen RJ, Griffin S (1985). The satisfaction with life scale. J Pers Assess.

[46] Arrindell WA, Heesink J, Feij JA (1999). The satisfaction with life scale (SWLS): appraisal with 1700 healthy young adults in the Netherlands. Personal Individ Differ.

[47] Collins CE, Boggess MM, Watson JF, Guest M, Duncanson K, Pezdirc K, Rollo M, Hutchesson MJ, Burrows TL. Reproducibility and comparative validity of a food frequency questionnaire for Australian adults. Clin Nutr. 2013;33(5):906–14. 10.1016/j.clnu.2013.09.015 24144913

[48] Godin G, Shephard R (1997). Godin leisure-time exercise questionnaire. Med Sci Sports Exerc.

[49] Bush K, Kivlahan DR, McDonell MB, Fihn SD, Bradley KA (1998). The AUDIT alcohol consumption questions (AUDIT-C): an effective brief screening test for problem drinking. Arch Intern Med.

[50] Antony MM, Bieling PJ, Cox BJ, Enns MW, Swinson RP (1998). Psychometric properties of the 42-item and 21-item versions of the depression anxiety stress scales in clinical groups and a community sample. Psychol Assess.

[51] Lamers S, Westerhof GJ, Bohlmeijer ET, ten Klooster PM, Keyes CL (2011). Evaluating the psychometric properties of the mental health continuum‐short form (MHC‐SF). J Clin Psychol.

[52] Mick E, Faraone SV, Spencer T, Zhang HF, Biederman J (2008). Assessing the validity of the quality of life enjoyment and satisfaction questionnaire—short form in adults with ADHD. J Atten Disord.

[53] Lancaster GA (2015). Pilot and feasibility studies come of age!. Pilot Feasibility Studies.

[54] Shanyinde M, Pickering RM, Weatherall M (2011). Questions asked and answered in pilot and feasibility randomized controlled trials. BMC Med Res Methodol.

[55] Cohen J. Statistical power analysis for the behavior science. 2nd Ed. Hillsdale, New Jersey: Lawrance Eribaum Association; 1988.

[56] Misan G, Oosterbroek C (2014). Practitioners’ guide to effective Men’s health messaging: Men’s health resource Kit 2.

[57] Patel MX, Doku V, Tennakoon L (2003). Challenges in recruitment of research participants. Adv Psychiatr Treat.

[58] Richards J, Hillsdon M, Thorogood M, Foster C. Face-to-face interventions for promoting physical activity. Cochrane Database Syst Rev. 2013;9:1–86. 10.1002/14651858.CD010392.pub2 PMC11542891 24085592

[59] Pignone MP, Ammerman A, Fernandez L, Orleans CT, Pender N, Woolf S, Lohr KN, Sutton S (2003). Counseling to promote a healthy diet in adults: a summary of the evidence for the US Preventive Services Task Force. Am J Prev Med.

[60] ACMA (2012). Communications report 2011–12 series. Report 2—Australia’s progress in the digital economy: participation, trust and confidence.

[61] Partridge SR, Allman-Farinelli M, McGeechan K, Balestracci K, Wong AT, Hebden L, Harris MF, Bauman A, Phongsavan P (2016). Process evaluation of TXT2BFiT: a multi-component mHealth randomised controlled trial to prevent weight gain in young adults. Int J Behav Nutr Phys Act.

[62] Vandelanotte C, Spathonis KM, Eakin EG, Owen N (2007). Website-delivered physical activity interventions: A review of the literature. Am J Prev Med.

[63] Gilson ND, Pavey TG, Vandelanotte C, Duncan MJ, Gomersall SR, Trost SG, Brown WJ. Chronic disease risks and use of a smartphone application during a physical activity and dietary intervention in Australian truck drivers. Aust N Z J Public Health. 2015;40(1):91–3. 10.1111/1753-6405.12501 26713400

[64] Internet User Demographics [http://www.pewinternet.org/data-trend/internet-use/latest-stats/]. Accessed 4 Mar 2016.

[65] Morgan PJ, Warren JM, Lubans DR, Collins CE, Callister R (2011). Engaging men in weight loss: experiences of men who participated in the male only SHED-IT pilot study. Obes Res Clin Prac.

[66] Godino JG, Merchant G, Norman GJ, Donohue MC, Marshall SJ, Fowler JH, Calfas KJ, Huang JS, Rock CL, Griswold WG (2016). Using social and mobile tools for weight loss in overweight and obese young adults (Project SMART): a 2 year, parallel-group, randomised, controlled trial. Lancet Diabetes Endocrinol.

[67] Yardley L, Spring B, Riper H, Morrison L, Crane D, Curtis K, Merchant G, Naughton F, Blanford A. Understanding and promoting effective engagement with digital behavior change interventions. Am J Prev Med. 2016;51(5):1–28. 10.1016/j.amepre.2016.06.015 27745683

[68] Hooker SP, Harmon B, Burroughs EL, Rheaume CE, Wilcox S. Exploring the feasibility of a physical activity intervention for midlife African American men. Health Educ Res. 2011;26(4):732–38. 10.1093/her/cyr034 PMC3139490 21597100

[69] O’Brien KM, Hutchesson MJ, Jensen M, Morgan P, Callister R, Collins CE (2014). Participants in an online weight loss program can improve diet quality during weight loss: a randomized controlled trial. Nutr J.

[70] Bravata DM, Smith-Spangler C, Sundaram V, Gienger AL, Lin N, Lewis R, Stave CD, Olkin I, Sirard JR (2007). Using pedometers to increase physical activity and improve health: a systematic review. JAMA.

[71] Abouserie R (1994). Sources and levels of stress in relation to locus of control and self esteem in university students. Educ Psychol.

[72] Waters L, St George A, Chey T, Bauman A (2012). Weight change in control group participants in behavioural weight loss interventions: a systematic review and meta-regression study. BMC Med Res Methodol.

[73] Johnson TP, O'Rourke DP, Burris JE, Warnecke RB (2005). An investigation of the effects of social desirability on the validity of self-reports of cancer screening behaviors. Med Care.

[74] Recommended number of serves for adults [https://www.eatforhealth.gov.au/food-essentials/how-much-do-we-need-each-day/recommended-number-serves-adults]. Accessed 7 Nov 2015.

