# Supplementary material for: Feasibility and preliminary efficacy of the ‘HEYMAN’ healthy lifestyle program for young men: a pilot randomised controlled trial
Source: Nutr J. 2017 Jan 13;16:2. doi: 10.1186/s12937-017-0227-8 (PMC5237246; doi:10.1186/s12937-017-0227-8)
Supplement: Additional file 1: — Full list of HEYMAN components and alignment to participatory responses and behaviour change strategies. (DOCX 24 kb) [file 12937_2017_227_MOESM1_ESM.docx]

| **Supplementary table 1: Full list of HEYMAN components and alignment to participatory responses and behaviour change strategies** | | | | | |
| --- | --- | --- | --- | --- | --- |
| **Components** | **Description of component** | **Recommended frequency of use** | **Participatory responses addressed** | | **SCT and/or SDT strategy** |
|  |  |  | **Focus groups (1)** | **Online survey (2)** |  |
| ***1. Password protected responsive website*** | The moderated responsive website (i.e., the same content on all devices e.g. smartphone, tablet and computer that is resized or repositioned to increase usability) included information, resources, pre-recorded short videos (e.g. cooking videos and Gymstick™ instructions), and suggestions of smartphone apps for improving diet, increasing aerobic and resistance-based exercise, reducing alcohol intake and strategies for coping with stress. The website included links to the Facebook group and calendar which outlined upcoming face-to-face sessions. New material was added to the website on average every two weeks to enhance engagement.  Graphic design of the website reflected young men (e.g., images of young men) and male engagement strategies that have been successful in previous men’s health programs (e.g. use of humour and camaraderie) were integrated.  Accessed at [www.heymanprogram.com](http://www.heymanprogram.com) using a predefined password. | Once per week | **Content preferences: *‘****education and skill development’* - e.g. cooking videos featured on website and resistance training videos to assist in correct technique using the Gymstick™.  **Key motivators:** *improve health, complement sporting goals and improve appearance* – program messages on the website focussed on the benefits of eating healthy and exercise on improving these motivating factors  **Key Barriers**: Offset *cost and lack of time barriers* with website tabs including ‘quick, easy and cheap healthy meals’ and ‘fun, free and local exercise’ | **Delivery mode:** Website was most preferred delivery mode for healthy eating components (55.1%) and third most favoured for exercise (30.1%) and stress (30.5%)  **Risk behaviours:** The top four risk behaviours that young men confirmed they needed more help to manage are all included on the website: unhealthy eating habits, not enough exercise, mental health problems and alcohol use.  **Key barriers**: 55% ranked “*lack of time to cook/ prepare healthy foods”* and 58% ranked *“lack of time to exercise”* as one of their top barriers. Also 42% ranked *cost of healthy foods* and 33% ranked *high cost of exercise equipment* as top barriers. The website offers information to offset these prominent barriers | *Prompt barrier identification* (SCT).  *Facilitation/Behavioural Capability* (SCT) (e.g. providing tools/ resources to make new behaviours easier to perform).  *Outcome Expectations (SCT):* (e.g. demonstrate positive outcomes from desired behaviour) |
| ***2. Wearable fitness band (JAWBONE™) and associated UP mobile app:*** | Worn 24 hours per day for the duration of the trial and associated fitness tracker app which assisted in goal setting and self-monitoring physical activity, diet, sleep and mood. | 24 hours per day | **Content preferences:** *Regular individualised progress/ feedback -*the UPapp automatically synced to allow participants to self-monitor behaviour and see progress. Smart notifications from the app also gave tips/ ideas to improve number of steps and sleep patterns. Also the app sent encouraging/motivational messages if met daily goals  **Content preferences:** *Realistic, individualised and flexible goals* – participants could set step and sleep goals. These were reviewed in week 3 during the one-to-one session and amended if too easy or too difficult to achieve. | **Delivery mode:** mobile apps was second most preferred delivery mode for healthy eating components (38.4%)  **Key Barrier:** 66.3% ranked ‘*lack of motivation’* as one of top barriers – offering incentives such as Jawbone may extrinsically motivate.  **Key Motivator:** 32% ranked *“to have more energy as a key motivator”* – by adhering to sleep and exercise goals on the app may help to improve daily energy levels. | *Self-regulation (SCT):* i.e. self-monitoring, goal setting & feedback. |
| ***3. Face-to-face sessions:*** | Weekly (1 hour) face-to-face sessions. There were 11 group based sessions and 1 individualised (one-to-one) which occurred in week three. The group based sessions took place on Thursday nights from 18:00-19:00pm for 10 out of the 11 sessions. One group based session (week 12) took place on Saturday morning (08:00am) to undertake a 5km ParkRun.  The group based session predominantly covered exercise (ran by Male PE teacher) with discussions on healthy eating and stress at the end with directions to components on website. | Once per week (60-minute session) | **Delivery mode preferences:** face-to-face sessions to include both individual and group based sessions  **Key Motivators:** group based exercise session looked to enhance *social inclusion, improve fitness, improve physical and mental health*  **Key Barrier:** Encouragement can enhance self-efficacy and offset *cognitive-emotional barriers* (e.g. feelings of inferiority and embarrassment) | **Delivery mode:** Face-to-face in a group was the most preferred delivery method for stress components (42.6%) and second most preferred for exercise components (47.9%). Face-to-face in a one-to-one setting was most preferred delivery method for exercise (54.6%%) and second most preferred for stress components (41.5%).  **Intervention preference:** A median of 4 face-to-face sessions per month was preferred | *Autonomy (SDT)* (e.g. providing choice – what exercises do you want to include in the circuit?). *Collective efficacy* (SCT) & *Relatedness* (SDT) – group based sessions to allow individuals to support each other and increase confidence.  *Vicarious experience* (SCT) – Use of male-only facilitators  *Verbal persuasion* (SCT)- encouragement and positive reinforcement throughout. |
| ***4.Personalised food and nutrient report*** | Participants completed a survey on their eating habits online. A feedback report was then generated which compares usual dietary intake to Australian dietary recommendations. The report was provided to participants and discussed in a 60-minute one-on-one, face-to-face session in week 3 and tailored dietary goals set in the session. The feedback report with the recommended set goals were then emailed to the participant immediately after the one-to-one session. | One 60-minute session in week 3 to discuss AES report and set personalised goals | **Content preferences:**  *individualised progress/ feedback-* the dietary report was personalised and based on the participant’s actual intake from the past 6 months  **Content preferences:** *Realistic, individualised and flexible goals -*Goals were set by the participants themselves with help from the facilitator. Participants were asked to speak with facilitators in upcoming group sessions if goals were difficult to achieve and amend if necessary. | **Risk behaviours:** 63% stated they needed more help managing unhealthy eating habits. | *Self-regulation (SCT)*: i.e. goal setting & feedback.  *Self-efficacy (SCT)*: Break down behaviour change into small, measurable steps |
| ***5. Private Facebook group:*** | Allowed participants to interact with other participants and the research team with any queries/questions. Links to new website material were posted on the Facebook page and events were created for upcoming face-to-face sessions. | Weekly | **Key Motivators:** *Social inclusion e.g. making friends* | **Key barriers:** 66% ranked ‘*lack of motivation to exercise*’ and 51% ranked “*lack of motivation to cook healthy foods”* as one of the key barriers. – The social support provided from other participants and the research team can assist in motivating the participants to perform the desired behaviours. | *Relatedness* *(SDT)* (e.g. social support from other male participants) |
| ***6. Gymstick™*** | Resistance training band allows home-based strength training (with associated routines available on the website). Also Gymstick routines targeting the different muscle groups was covered in the face-to-face sessions. | 2 days per week in line Australian physical activity guidelines which recommend muscle strengthening activities on at least 2 days each week | **Content preference:** *Gradual build-up of fitness and strength*  **Key motivators**: *To improve body image-* by improving muscle mass the young men may improve their self-perception of appearance.  **Key barrier:** the home-based workout can offset *cognitive emotional barriers* expressed were associated with going to the gym (e.g. feelings of inferiority and embarrassment) | **Key barriers:** 66% ranked ‘*lack of motivation’* as one of the top barriers – offering incentives such as Gymstick may extrinsically motivate. Also 33% ranked “*high cost of equipment/ facilities”* as one of the top barriers. Since Gymstick can do a number of exercise routines at home, participants will save money on gym memberships / gym equipment.  Furthermore, 58% ranked *“lack of time to exercise”* as any one of top barriers – the home based workout can save time in travelling to and from the gym.  **Key motivator:** 45% ranked ‘*improve body image’* as any one of top motivators – resistance training has the potential to increase muscle mass and may increase self-perception of appearance | *Competence* (SDT); introduce activities and allow participants to do at home to master skill  *Facilitation/Behavioural Capability* (SCT) (e.g. providing tools/ resources to make new behaviours easier to perform). |
| ***7. TEMPlate™ dinner disc:*** | Simple visual guide to assists in controlling the portion size of foods. | Every day with main meals | **Content preferences:** *education and skill development* – e.g. inform of correct portion sizes of different food groups on dinner plate | **Key Barrier:** 51% ranked *“lack of motivation to cook healthy foods”* as any one of the top barriers. – Providing participants with this tool kit may motivate them to cook and prepare healthier meals. | *Facilitation/Behavioural Capability* (SCT) (e.g. providing tools/ resources to make new behaviours easier to perform) |

1. Ashton LM, Hutchesson MJ, Rollo ME, Morgan PJ, Thompson DI, Collins CE. Young adult males’ motivators and perceived barriers towards eating healthily and being active: a qualitative study. International Journal of Behavioral Nutrition and Physical Activity. 2015;12(1):93.

2. Ashton L, Hutchesson, M., Rollo, M. Morgan, P., Collins, C. Motivators and barriers to engaging in healthy eating and physical activity: A cross-sectional survey in young adult men. Manuscript submitted for publication 2016.
