# Supplementary material for: Feasibility and preliminary efficacy of the ‘HEYMAN’ healthy lifestyle program for young men: a pilot randomised controlled trial
Source: Nutr J. 2017 Jan 13;16:2. doi: 10.1186/s12937-017-0227-8 (PMC5237246; doi:10.1186/s12937-017-0227-8)
Supplement: Additional file 2: — Mean change in outcomes within groups and differences between groups (Intention-to-Treat Populations) at 3 months - results with follow-up data for the withdrawn participant for outcomes effected by medication. (DOCX 14 kb) [file 12937_2017_227_MOESM2_ESM.docx]

| **Supplementary table 2:**  **Mean change in outcomes within groups and differences between groups (Intention-to-Treat Populations) at 3 months - results with follow-up data for the withdrawn participant for outcomes effected by medication.** | | | | | |
| --- | --- | --- | --- | --- | --- |
|  | **Mean change from baseline (95%CI)^a^** | |  |  |  |
| **Outcomes^C^** | **Control group (n=24)** | **Intervention group (n=26)** | **Mean difference between groups (95%CI)^b^** | **p-Value** | **Effect size (Cohen’s *d*)** |
| Weight (kg) | 1.0 (-0.1, 2.2) | -0.1 (-1.2, 1.0) | -1.2 (-2.7, 0.4) | 0.157 | 0.41 |
| Weight (%) | 1.3 (0.1, 2.6) | -0.2 (-1.4, 1.0) | -1.5 (-3.3, 0.2) | 0.087 | 0.46 |
| Waist circumference (cm) | 1.9 (0.5, 3.3) | -0.7 (-2.1, 0.6) | -2.6 (-4.5, -0.7) | **<0.01** | 0.69 |
| BMI (Kg/m^2^) | 0.3 (-0.0, 0.7) | -0.1 (-0.4, 0.2) | -0.4 (-0.9, 0.1) | 0.101 | 0.46 |
| Body fat mass (kg) | 0.9 (-0.2, 1.9) | -0.0 (-1.0, 1.0) | -0.9 (-2.3, 0.5) | 0.213 | 0.34 |
| Total cholesterol (mmol/l) | 0.0 (-0.2, 0.3) | -0.3 (-0.6, -0.1) | -0.3 (-0.7, 0.0) | 0.062 | 0.44 |
| HDL-Cholesterol (mmol/l) | 0.0 (-0.1, 0.1) | 0.1 (-0.0, 0.1) | 0.0 (-0.1, 0.1) | 0.621 | 0.00 |
| LDL- Cholesterol (mmol/l) | 0.1 (-0.1, 0.4) | -0.3 (-0.5, -0.1) | -0.4 (-0.7, -0.1) | **<0.01** | 0.66 |
| Triglyceride (mmol/l) | -0.2 (-0.5, -0.0) | -0.0 (-0.3, 0.2) | 0.2 (-0.1, 0.6) | 0.224 | 0.32 |
| Total cholesterol / HDL-C ratio | -0.0 (-0.2, 0.2) | -0.4 (-0.6, -0.2) | -0.3 (-0.6, -0.1) | **<0.05** | 0.59 |
| Systolic blood pressure (mm Hg) | -2.6 (-5.7, 0.5) | -1.5 (-4.5, 1.4) | 1.1 (-3.2, 5.4) | 0.610 | 0.14 |
| Diastolic blood pressure (mm Hg) | -1.1 (-2.9, 0.8) | -1.3 (-3.1, 0.5) | -0.2 (-2.8, 2.3) | 0.858 | 0.04 |
| ^a^Time differences were calculated as (3 month – baseline).  ^b^Between group differences at 3 months  ^c^ Adjusted for baseline values of BMI, physical activity steps and proportion of energy from Energy-dense, Nutrient poor (ED-NP) foods  Abbreviations: ARFS: Australian Recommended Food Score, ED-NP: Energy-Dense, Nutrient poor, HDL: High Density Lipoprotein, LDL: Low Density Lipoprotein | | | | | |
