# Supplementary material for: Feasibility and preliminary efficacy of the ‘HEYMAN’ healthy lifestyle program for young men: a pilot randomised controlled trial
Source: Nutr J. 2017 Jan 13;16:2. doi: 10.1186/s12937-017-0227-8 (PMC5237246; doi:10.1186/s12937-017-0227-8)
Supplement: Additional file 3: — Mean change in subscales of the ARFS within groups and differences between groups (Intention-to-Treat Populations) at 3 months. (DOCX 13 kb) [file 12937_2017_227_MOESM3_ESM.docx]

| **Supplementary table 3: Mean change in subscales of the ARFS within groups and differences between groups (Intention-to-Treat Populations) at 3 months.** | | | | | |
| --- | --- | --- | --- | --- | --- |
|  | **Mean change from baseline (95%CI)^a^** | |  |  |  |
| **Outcomes^C^** | **Control group (n=24)** | **Intervention group (n=26)** | **Mean difference between groups (95%CI)^b^** | **p-Value** | **Effect size (Cohen’s *d*)** |
| **Diet quality** |  |  |  |  |  |
| ARFS Vegetables | 0.9 (-0.7, 2.4) | 2.7 (1.2, 4.3) | 1.9 (-3.3, 4.1) | 0.095 | 0.46 |
| ARFS Fruit | 0.0 (-1.1, 1.2) | 1.8 (0.7, 3.0) | 1.8 (0.1, 3.4) | **<0.05** | 0.58 |
| ARFS Meat | 0.1 (-0.3, 0.6) | 0.4 (-0.1, 0.8) | 0.2 (-0.4, 0.9) | 0.460 | 0.18 |
| ARFS Vegetarian alternatives | 0.5 (-0.0, 1.0) | 0.3 (-0.2, 0.8) | -0.2 (-0.8, 0.5) | 0.642 | 0.15 |
| ARFS Wholegrains | 0.9 (-0.2, 2.0) | 0.7 (-0.3, 1.8) | -0.2 (-1.7, 1.3) | 0.814 | 0.07 |
| ARFS Dairy | -0.0 (-0.6, 0.5) | -0.0 (-0.6, 0.5) | -0.0 (-0.8, 0.8) | 0.997 | 0.00 |
| ARFS Water | -0.1 (-0.3, 0.1) | 0.2 (0.0, 0.4) | 0.3 (0.1, 0.5) | **<0.05** | 0.71 |
| ^a^Time differences were calculated as (3 months – baseline).  ^b^Between group differences at 3 months  ^c^ Adjusted for baseline values of BMI, physical activity steps and proportion of energy from Energy-dense, Nutrient poor (ED-NP) foods  Abbreviations: ARFS: Australian Recommended Food Score | | | | | |
